# A Vision Transformer Model for the Prediction of Fatal Arrhythmic Events in Patients with Brugada Syndrome

**DOI:** 10.3390/s25030824

**Published:** 2025-01-30

**Authors:** Vincenzo Randazzo, Silvia Caligari, Eros Pasero, Carla Giustetto, Andrea Saglietto, William Bertarello, Amir Averbuch, Mira Marcus-Kalish, Valery Zheludev, Fiorenzo Gaita

**Affiliations:** 1 Department of Electronics and Telecommunications (DET), Politecnico di Torino, 10129 Turin, Italy; 2 Division of Cardiology, Città della Salute e della Scienza Hospital, 10126 Turin, Italy; 3 Department of Medical Sciences, University of Turin, 10124 Turin, Italy; 4 School of Computer Science, Tel Aviv University, Tel Aviv 6997801, Israel; 5 Department of Statistics and Operations, Tel Aviv University, Tel Aviv 6997801, Israel; 6 J Medical, Cardiology Unit, 10151 Turin, Italy

**Keywords:** deep learning, vision transformer, Brugada syndrome, electrocardiogram, risk stratification, sudden cardiac death

## Abstract

Brugada syndrome (BrS) is an inherited electrical cardiac disorder that is associated with a higher risk of ventricular fibrillation (VF) and sudden cardiac death (SCD) in patients without structural heart disease. The diagnosis is based on the documentation of the typical pattern in the electrocardiogram (ECG) characterized by a J-point elevation of ≥2 mm, coved-type ST-segment elevation, and negative T wave in one or more right precordial leads, called type 1 Brugada ECG. Risk stratification is particularly difficult in asymptomatic cases. Patients who have experienced documented VF are generally recommended to receive an implantable cardioverter defibrillator to lower the likelihood of sudden death due to recurrent episodes. However, for asymptomatic individuals, the most appropriate course of action remains uncertain. Accurate risk prediction is critical to avoiding premature deaths and unnecessary treatments. Due to the challenges associated with experimental research on human cardiac tissue, alternative techniques such as computational modeling and deep learning-based artificial intelligence (AI) are becoming increasingly important. This study introduces a vision transformer (ViT) model that leverages 12-lead ECG images to predict potentially fatal arrhythmic events in BrS patients. This dataset includes a total of 278 ECGs, belonging to 210 patients which have been diagnosed with Brugada syndrome, and it is split into two classes: *event* and *no event*. The *event* class contains 94 ECGs of patients with documented ventricular tachycardia, ventricular fibrillation, or sudden cardiac death, while the *no event* class is composed of 184 ECGs used as the control group. At first, the ViT is trained on a balanced dataset, achieving satisfactory results (89% accuracy, 94% specificity, 84% sensitivity, and 89% F1-score). Then, the discarded *no event* ECGs are attached to additional 30 event ECGs, extracted by a 24 h recording of a singular individual, composing a new test set. Finally, the use of an optimized classification threshold improves the predictions on an unbalanced set of data (74% accuracy, 95% negative predictive value, and 90% sensitivity), suggesting that the ECG signal can reveal key information for the risk stratification of patients with Brugada syndrome.

## 1. Introduction

Since its introduction as a new clinical entity by Pedro and Josep Brugada in 1992 [1], the Brugada syndrome (BrS) has attracted great interest because of its high prevalence in many parts of the world and its association with an increased risk of sudden death in young people without any structural heart disease. Recent years have witnessed a dramatic rise in the number of reported cases and a great proliferation of papers serving to define the clinical, genetic, and molecular aspects of the disease [2,3]. Ten percent of the patients whose electrocardiogram (ECG) shows the typical BrS pattern is symptomatic for cardiac arrest or syncope, often occurring at rest, especially after a large meal, or during sleep. Fever, alcohol, and medications may unmask the BrS pattern in asymptomatic patients and increase the risk of arrhythmic events [4,5].

The diagnosis is made by recording an electrocardiogram and identifying the distinctive pattern in the right precordial leads [6,7,8,9]. Sometimes only a suspicious pattern is present, in the past described as ‘type 2’ (≥2 mm J-point elevation, ≥1 mm ST-segment elevation with saddleback appearance, followed by a positive or biphasic T-wave) or ‘type 3’ (either a saddleback or coved-type ST-segment elevation <1 mm), and the administration of a sodium channel blocking drug may be necessary to reveal the ECG diagnostic pattern, if not spontaneously present [10].

Electrocardiography is the key tool for clinicians to detect potential arrhythmogenic conditions. The ECG is capable of recording the electrical activity of the heart by tracking the voltage changes in time during the cardiac cycle. In particular, the 12-lead ECG provides information on heart activity from six limb leads positioned on the arms and legs and six precordial leads which are placed in specific locations on the chest.

The use of artificial intelligence (AI) for ECG analysis has gained significant momentum in recent times since ECG is an ideal substrate for AI applications, being a widely adopted and cost-effective cardiological tool. Many recent types of research have reported positive results using AI-based ECG analysis in several clinical settings. Attia et al. developed an AI-enabled electrocardiogram analysis using a convolutional neural network to detect the electrocardiographic signature of atrial fibrillation present during normal sinus rhythm [11]. Furthermore, deep learning techniques with several neural network schemes are used to estimate arterial blood pressure starting from photoplethysmogram (PPG) and electrocardiogram signals providing for a noninvasive blood pressure measurement [12,13,14]. Convolutional neural models have been optimized and modified for the automatic detection and classification of arrhythmias [15,16] and, more generally, heartbeat classification from ECGs [17,18,19]. This recent progress has created an exciting possibility of employing AI ECG interpretation for the detection of Brugada syndrome. A recent work [20] has proposed a deep learning algorithm for the detection of Brugada features in ECGs to predict the response of the ajmaline drug test. In [21], an AI-enabled algorithm with a convolutional neural network was proposed with a recall of 0.77±0.14, a negative predictive value of 0.94±0.11, and a positive predictive value of 0.44±0.29. The Brugada Syndrome and Artificial Intelligence Applications to Diagnosis (BrAID) project [22] developed a system for diagnosing type 1 BrS through the application of machine learning (ML) algorithms using as input three leads (V1, V2, and V3) with a comparable accuracy where only V2 (80.20%) is taken into account. Moreover, a transfer learning technique has been proposed to identify the right bundle branch block pattern for transferring the information into a trained network to diagnose the type 1 Brugada ECG pattern, and the performance of the model has been compared with cardiologists, obtaining good results (sensitivity 86.0%, specificity 90.0%) [23].

Different risk scores have been proposed for the diagnosis and risk stratification of patients with a BrS pattern [24,25,26]. However, as per the recent work of Gaita et al., the main concern for clinicians is the risk stratification of BrS patients, i.e., to distinguish those who could develop a major arrhythmic event from those who will remain asymptomatic for the rest of their lives [27]. For this reason, it is worth investigating whether the use of AI could improve the risk stratification. Indeed, at this time, the only objective data available to identify patients at high risk of arrhythmia are clinical indicators, such as a history of aborted sudden death, unexplained syncope, previous occurrence of sustained ventricular tachycardia (VT), and a spontaneous type 1 ECG pattern [28]. The impact of a family history of sudden cardiac death (SCD) and positive genetic testing in risk stratification are still unresolved problems [29,30]. Genetic analysis focused on the correlation between the presence of genetic variants in the SCN5A gene and the occurrence of arrhythmic events, has not yet achieved substantial contributions [31,32]. Clinical, electrophysiological, and electrocardiographic markers have been introduced to improve risk stratification [33,34,35,36,37]: the latter was at first determined manually [38] and then evaluated through automatic measurement [39], decreasing the risk of variability and bias.

Creating an AI model for the detection and risk stratification of Brugada syndrome presents a significant challenge due to the rarity of patients with an arrhythmic event [27]. While AI-based ECG analysis has been used for the detection of Brugada syndrome, risk stratification remains challenging [40] and an interest is growing in using AI algorithms to predict fatal events in BrS patients. This approach may provide clinicians with an automatic tool to guide clinical decision making and prevent sudden cardiac death (SCD). A first attempt to find specific BrS ECG features for risk stratification is shown in [41], based on the idea that ECG may contain key information about the risk of developing arrhythmic events. However, the main drawback of this approach is the use of features manually extracted by expert cardiologists. On the contrary, a pure AI approach should be able to work directly on the ECGs, without the need for any prior cardiologist feature extraction phase.

This work tackles the limitation of using manually extracted features and proposes a novel AI-enhanced ECG analysis, based on the vision transformer (ViT) architecture, as a support for the recognition of patients at high risk of developing arrhythmias. ViT does not require any prior feature extraction phase but it works directly on the ECG scans; therefore, it is not subject to any human bias related to feature choice or their measurement. A total of 278 ECGs from the Piedmont Brugada registry are considered [42]. To be more specific, 94 ECGs belong to patients who developed arrhythmic events, while the remaining ones are the ECGs of patients without arrhythmic events. The proposed model does not need any pre-processing, and only a manual cropping for highlighting all the leads is applied, without any loss of information and expensive computational costs.

Section 2 briefly presents the main characteristic of Brugada syndrome, underlying the difficulties in diagnosis and risk stratification. In Section 3, the ECG dataset is described, showing the distribution of the population in the two classes (“*event*” and “*no event*”). Section 4 explains the architecture of the model taken into account (ViT) together with the use of the ROC curve for the identification of the optimized threshold for classification. In Section 5, all the results are collected, and Section 6 gives an analysis of the experiments. At last, Section 7 sums up the main contributions of this work.

## 2. Brugada Syndrome and Risk Stratification

**Figure 1:**
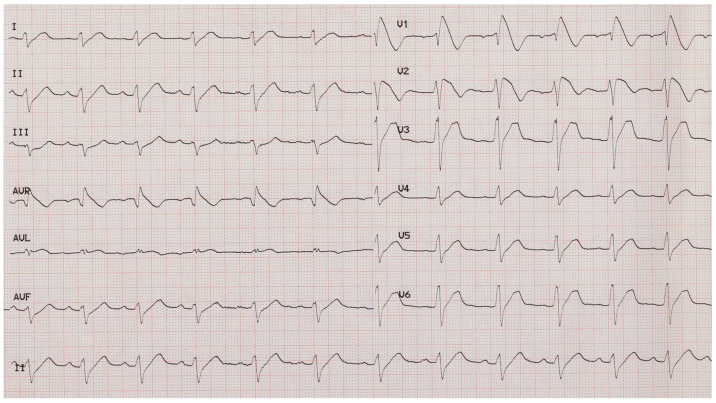
Example of an electrocardiogram showing the Brugada type 1 pattern.

Specific diagnostic criteria and three ECG patterns for Brugada syndrome were presented by a consensus report [10]: type 1 is characterized by ST-segment elevation ≥2 mm (0.2 mV) in at least one right precordial leads (V1–V3) positioned in the 4th, 3rd, or 2nd intercostal space, followed by a negative T wave, which represents the only electrocardiographic abnormality for potential diagnosis (see Figure 1). Type 2 (≥2 mm J-point elevation, ≥1 mm ST-segment elevation with saddleback appearance, followed by a positive T-wave) and type 3 (either a saddleback or coved-type ST-segment elevation <1 mm) are not diagnostic and are currently defined as suspicious patterns. In the presence of these suspicious patterns, especially if associated with a family history of SDC at less than 45 years old, syncope, and agonal respiration, patients should be subjected to pharmacological test with sodium-channel blocking drugs [43]. If a type 1 ECG pattern occurs during drug administration, the test is positive.

According to the 2022 version of the European Society of Cardiology guidelines, Brugada syndrome is diagnosed by the presence of spontaneous type 1 ECG regardless of symptoms, or in the presence of an drug-induced type 1 ECG associated with other clinical features [44], such as documented persistent ventricular tachycardia or ventricular fibrillation or arrhythmic syncope or relevant family history.

Among patients with Brugada ECG pattern, less than 20% of subjects are symptomatic. In this group, the therapeutic strategy is well defined. Generally, an implantable cardioverter defibrillator (ICD) is suggested [44].

The main concern regards asymptomatic individuals since they represent more than 80% of Brugada patients. In this group of patients, it is mandatory to identify those who will develop a major arrhythmic event, since cardiac arrest or sudden death mostly occur as the first symptoms of the disease.

In asymptomatic patients with a spontaneous type 1 ECG pattern, an electrophysiological (EP) study should be performed to identify higher-risk patients who need therapy [45]. However, even a negative result of the EP study does not completely exclude the occurrence of major arrhythmic events. In this sense, a system able to predict fatal arrhythmic events in asymptomatic patients is of great interest for the clinical community.

## 3. ECG Dataset

### 3.1. Study Population

In this study, 278 12-lead-ECGs were analyzed from the Piedmont Brugada registry [40,42], belonging to 210 patients with a diagnosis of Brugada syndrome. The acquisition time was 5 s, as per the medical standard paper ECG. Of the 148 patients classified as no event, 117 (79%) were male, with a mean age at the time of ECG of 43.6 years. Only 10 of them (6.76%) had a history of unexplained syncope; the majority had a negative clinical history or history of vasovagal syncope. Family history of SCD was present in 13 patients (8.78%). Moreover, 81 subjects underwent electrophysiological study, which was positive in 23 of them (28.4%). Finally, 64 patients underwent genetic testing for the SCN5A mutation, which was positive in 25 cases (30%).

Among the 62 patients classified as event, 89.4% are male, with an average age at the time of the ECG of 45.5 years. A history of unexplained syncope is present in 42.1%, while 13.1% have a positive family history for SCD. The electrophysiological study was performed in 52% of the subjects, with positivity found in 70%. Finally, 27 patients performed a search for the genetic mutation of SCN5A, which was positive in 44.4% (see Table 1).

The patients were divided into two groups, with one (*event*) including 94 ECGs belonging to 62 patients with a history of severe arrhythmic events (as defined later), and the other (*no event*) including 184 ECGs belonging to 148 patients without this type of episode. All the ECGs were taken from patients inscribed in the prospective Brugada Registry of the Piedmont region, Italy. All the patients had a documented type 1 Brugada ECG pattern. Patients who were taking the drug quinidine or hydroquinidine or who had undergone a substrate ablation were excluded from the analysis because these therapies reduce the risk of arrhythmic events.

### 3.2. Dataset Description

The dataset consists of 278 12-lead ECGs from the Piedmont Brugada registry [40,42], 94 of which belong to patients who developed arrhythmic events: sustained ventricular tachycardia (VT) or ventricular fibrillation (VF) or SCD (syncope was not included due to the difficulty in discerning from neurally mediated syncope, which is independent of the syndrome and generally benign). Most of the episodes of VF occurred in patients already implanted with an ICD. In the following, this class will be referred as the *event* class.

The remaining part (184 ECGs) includes the patients with spontaneous type 1 Brugada ECG but without arrhythmic events; in the following, this class will be referred as *no event*.

Due to the limited number of available ECGs, to guarantee the proper generalization of the findings, two datasets were created for the classification of ECGs starting from the above population, as defined subsequently: *Balanced Dataset* (94-94) and *Balanced Training Set and Unbalanced Test Set* (90-30). For the same purpose, k-fold cross-validation and leave-one-out techniques were employed.

Due to the imbalance between the two classes (94 vs. 184 ECGs), the first dataset, say *Balanced Dataset* (94-94) considers an equal number of *event* and *no event* samples, i.e., the *no event* class was randomly reduced to have the same cardinality as the *event* class, obtaining a balanced dataset (94 *events* against 94 *no events*). There appears to be no definitive rule regarding the optimal ratio for splitting a dataset into training and test sets. The commonly used Pareto principle (80:20) serves as a general guideline adopted by practitioners [46]. Additionally, the inclusion of a validation set allows for model evaluation prior to the testing phase. The size of the validation set, like those of the training and test sets, can vary depending on the specific needs of the analysis. In this work, a standard proportion was chosen to ensure a balanced approach while preserving as much data as possible for training, given the limited size of the dataset. Then, the dataset was split into a training set, consisting of around 70% of the entire dataset, a validation set (≈10%), and a test set (20%), maintaining the balance between the two classes (i.e., where each subset contains an equal number of *event* and *no event* data). The following notation will be adopted for identifying the training, validation, and test sets of this dataset:

- Training set, $TrS_{1}$: 136 ECGs (68 *events* and 68 *no events*);
- Validation set, $VlS_{1}$: 14 ECGs (7 *events* and 7 *no events*);
- Test set, $TsS_{1}$: 38 ECGs (19 *events* and 19 *no events*).

Due to the relatively small amount of ECGs and the strong unbalance between the two classes, the use of a balanced training set is justified to reduce classification biases; indeed, with a few data, the risk is that ViT would only focus on the class relative distribution and use it to perform risk stratification instead of extracting additional information from the input ECGs to discriminate between the *event* and *no event* groups. Furthermore, since the test set is not used during the training procedure, i.e., during the tuning of the technique, it constitutes an independent, external set of patients, employed to assess the generalizability of the findings.

Moving forward with this approach, the second dataset still considers balanced training and validation sets; however, in this case, to better mimic the real distribution of symptomatic patients, it was chosen to use an unbalanced test set. The training ($TrS_{2}$) and validation ($VlS_{2}$) sets were derived from the *Balanced Dataset* (94-94), considering 90% and 10%, respectively. The test set ($TsS_{2}$) was composed of the unused *no event* ECGs (90) together with 30 *event* ECGs, extracted from a single individual 24-h Holter. The full dataset will be referred as *Balanced Training Set and Unbalanced Test Set* (90-30), whose three subsets are constituted as follows:

- Training set, $TrS_{2}$: 170 ECGs (85 *events* and 85 *no events*);
- Validation set, $VlS_{2}$: 18 ECGs (9 *events* and 9 *no events*);
- Test set, $TsS_{2}$: 120 ECGs (30 *events* and 90 *no events*).

## 4. Method

**Figure 2:**
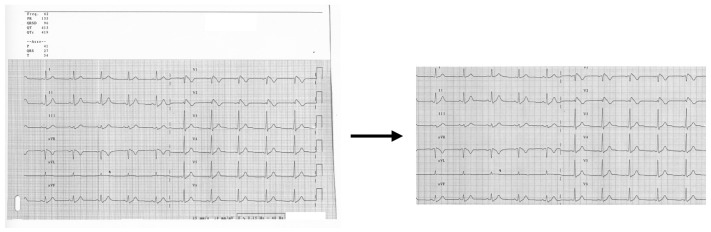
Original ECG (on the **left**) and manual cropping of ECG (on the **right**) used as input for the model.

The proposed models do not need specific pre-processing techniques. Only manual cropping is required to remove the heading and footer information (e.g., sex, age, ECG intervals, and so on), where present. This approach allows one to highlight all 12-lead in ECGs, without the risk of losing information and complex computations (see Figure 2).

### 4.1. Vision Transformer

The Vision Transformer (ViT) is a model for image classification inspired by the Transformer [47], a simple network architecture based on attention mechanisms, and recently emerged as an alternative to convolutional neural networks (CNNs).

Proposed for the first time in [48], vision transformers have already been applied in various fields, including healthcare: deep learning pipeline based on ViT were proposed for gastric histopathological image detection, enabling the automatic global detection of gastric cancer images [49]; for the automatic recognition of tuberculosis from chest X-ray imaging [50]; for the classification of emphysema subtypes via CT images [51]; and, more recently, for the risk stratification of short QT patients [52]. Therefore, the ViT has proven to be an efficient technique to deal with medical images in improving clinical diagnoses and it has been chosen as the tool to address the Brugada risk stratification task. This approach is particularly valuable as vision transformers represent a relatively recent advancement in deep learning showing great promise in domains traditionally dominated by convolutional neural networks. Additionally, risk stratification in Brugada patients is an area still surrounded by significant uncertainties and limited exploration, making it even more compelling to address through machine learning models. By adopting ViT, this work contributes to developing practical, data-driven tools providing new insights into an area with a limited precedent.

A standard transformer architecture receives as input a one-dimensional sequence of tokens. Then, the main procedure introduced by ViT is to split an image into a sequence of two-dimensional fixed-size patches which are flattened and mapped to a 1D constant vector with a trainable linear projection obtaining the correct dimensionality for input into the transformer. The outputs are referred to as embedded patches.

**Figure 3:**
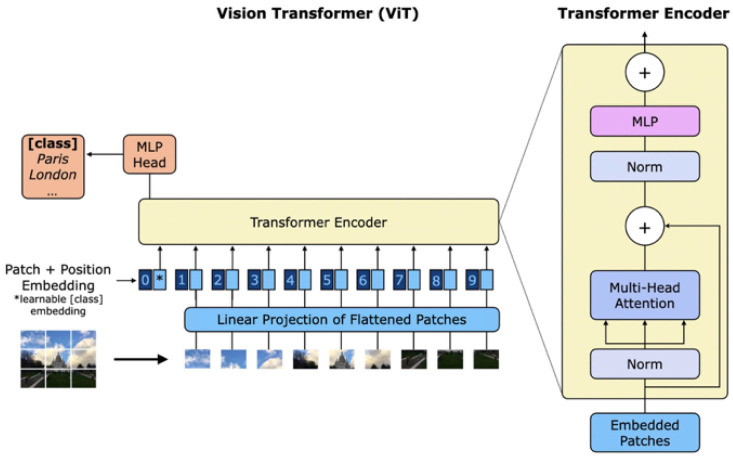
An overview of the vision transformer architecture and detailed representation of transformer encoder’s structure.

Because transformers do not have any mechanism that takes into account the order of patch embeddings, positional embeddings, learned vectors with the same dimension of patch embeddings, are added to retain positional information. The resulting tokens are inserted into the transformer encoder which consists of normalization layers, several parallel multi-head attention operations, a multilayer perceptron, and residual connections. To perform classification, an extra learnable classification token is added to the sequence. An overview of the model is represented in Figure 3.

The model was implemented in the Python programming language and the HuggingFace Hub Transformer was adopted for preprocessing the images and fine-tuning a pre-trained Vit network [53,54].

Vision Transformer model was pre-trained on ImageNet-21k at a resolution of 224 × 224 pixels and images were presented as a sequence of fixed-size patches 16 × 16 and normalized across the RGB channel with mean (0.5, 0.5, 0.5) and standard deviation (0.5, 0.5, 0.5).

All the proposed models were trained with a batch size of 32, a learning rate equal to $2\cdot 10^{-5}$, a warm-up ratio of 0.1, and a weight decay of 0.1.

### 4.2. Metrics

To compare the different models, six different metrics were evaluated from the confusion matrices:

- *Accuracy*: it is the ratio of correct classification to the total number of samples.
- *Positive predictive value (PPV) or precision*: it is the fraction of correctly positive predicted samples over the total positive predicted ones.
- *Negative predictive value (NPV)*: it is the probability that a negative predicted sample is effectively negative labeled.
- *Sensitivity or recall*: it is the fraction of correctly positive predicted samples over the cardinality of the positive class.
- *Specificity*: it is the fraction of true negatives samples that are correctly identified by the model.
- *F1-score*: it is a harmonic mean of the precision and recall, where the contributions of the two metrics are equal.

In the above, positive and negative classes refer to *event* and *no event* groups, respectively.

### 4.3. Optimized Threshold

Even though all the models were trained on a balanced dataset, true positive predictions are more important from a clinical point of view, since they refer to patients who will face an arrhythmic event.

At this purpose, in some experiments (see Section 5.2 and Section 5.3), the ROC curve was used for evaluating the prediction scores on the validation set to find the best classification threshold to maximize both specificity and sensitivity metrics.

In the following, the definitions of false positive rate (*FPR*) and true positive rate (*TPR*) in binary classification with regard to the confusion matrix notation (here, *TP* := true positives, *FP*:= false positives, *FN*:= false negatives, *TN*:= true negatives) are used.

The false positive rate is the fraction of negative samples that are incorrectly predicted with positive labels:

(1) $$FPR:=\frac{FP}{FP+TN}$$

The true positive rate is an alternative way of referring to sensitivity, and thus, it is computed as:

(2) $$TPR:=\frac{TP}{TP+FN}$$

The ROC curve is a plot representing the true positive rate (on the Y axis) against the false positive rate (on the X axis) for different classification thresholds. Usually, a sample is predicted to belong to the positive class if its probability (*softmax score*) is greater than 0.5. However, this threshold may be chosen between 0 and 1.

**Figure 4:**
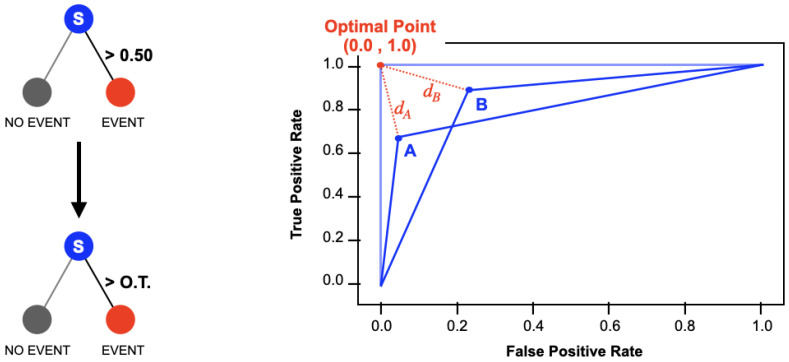
Schematic representation of the processes involved in the classification with the optimized threshold (O.T.): the classification procedure depending on the new threshold (**left**) and the evaluation of the distance from the ideal point to the ROC curve point (**right**).

The ideal point in the ROC curve is located in the top left corner, with an FPR equal to 0 and a TPR equal to 1 (see Figure 4), and it represents the best performance that a model could achieve. In order to minimize the amount of ECG misclassifications, the value of the classification threshold with the minimal distance (*d*) between points on the ROC curve and the ideal point was selected:

(3) $$d=\sqrt{FPR^{2}+{\left(1-TPR\right)}^{2}}$$

## 5. Results

In this section, the results of four different testing scenarios (*Test1*, *Test1.1*, *Test2*, *Test3*) are presented. *Test1* considered the *Balanced Dataset* (94-94), where the 10-fold cross-validation and the leave-one-out technique were also investigated as validation procedures. In *Test1.1*, a set of neural network models, generated in the previous experiment, was collected to assess their generalization capabilities on a new set of data, specifically the unbalanced test set $TsS_{2}$. Then, the *Balanced Training Set and Unbalanced Test Set* (90-30) dataset was adopted in *Test2* and *Test3*, where the accuracy and the F1-score were considered metrics for the validation process, respectively. In addition, the early stopping technique and the choice of an optimized classification threshold (as defined in Section 4.3) were included, monitoring the performance of the models on the validation test $VlS_{2}$, i.e., the train is stopped when the performance on the validation set has not improved according to the defined metric (accuracy in *Test2* and F1-score in *Test3*) after a number of iterations equal to 10.

Several combinations of hyper-parameters were evaluated for training; in the end, the learning rate, the warmup ratio, and the weight decay were set equal to $2\cdot 10^{-5}$, 0.1, and 0.1, respectively. These values were maintained for all the following experiments.

### 5.1. Test1: Balanced Dataset (94-94)

A total of 22 experiments were computed on the *Balanced Dataset* (94-94), which was split into the training set (70%), composed of 136 ECGs (68 events and 68 no events); the validation set (10%), composed of 14 ECGs (7 events and 7 no events); and the training set (20%), composed of 38 ECGs (19 events and 19 no events).

The models were trained for 40 epochs and, at end of each training, the model with the best accuracy obtained on the validation set, say *bestNN_Test1*, was saved and used for testing. The choice of accuracy instead of ROC-AUC metric lies on the fact that the two metrics, for the classical binary classification of balanced datasets, are identical (see Appendix A). Table 2 shows the means and standard deviations of the most significant metrics for the *bestNN_Test1* models on test set $TsS_{1}$, where all metrics are above 0.80 (with accuracy at 0.89, PPV at 0.94, and specificity at 0.94, all highlighted in bold).

A 10-fold cross-validation was performed to verify the performance of the previous experiments. K-fold cross-validation is a common practice for supervised machine learning experiments where the dataset is randomly partitioned into *k* subsets. The model is trained on k−1 partitions and the remaining part is used as the test set for validation. The procedure is iterated by shifting the test set to a different partition of the dataset in order to evaluate all the data. A stratified splitting of the entire dataset was applied for all the iterations to maintain the exact proportion between classes. In addition, the leave-one-out procedure (*k*-fold cross-validation with k=N, where *N* is the number of data) was performed to obtain an accurate estimation of the model capability. Even though the dataset contains more than one ECG per patient, each ECG record represents a unique description of the electrical cardiac signal. Indeed, Brugada is a dynamic syndrome whose pattern is not always present in the ECG of a specific patient; furthermore, the influence on the k-fold cross-validation or leave-one-out results is mitigated since the ratio is roughly 1.3 ECG per patient. Table 3 reports the performance comparison among the average results of the 22 experiments (from Table 2), the 10-fold cross-validation, and the leave-one-out. It can be observed that accuracy, PPV, and specificity slightly decrease compared to Table 2, but still exhibit promising classification performances.

#### Test1.1: Unbalanced Test Set Holter ECGs

The main relevant aspect of the previous scenario (*Test1* with the *Balanced Dataset*) is the high performance achieved. However, to better investigate model generalization capabilities, a second test set, $TsS_{2}$, was introduced. For these experiments, an optimal number of models generated by training on the $TrS_{1}$ dataset was taken into account, so that they were able to sum up the average behavior of the 22 experiments computed in *Test1* (as can be seen in Table 2). The average results of the predictions on the ECG scans of $TsS_{2}$ are presented in Table 4.

**Figure 5:**
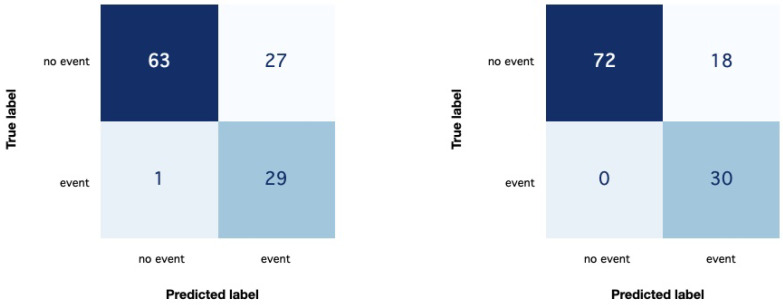
The confusion matrices of two different models of *Test1.1* on THE $TsS_{2}$ test set, whose accuracy is equal to 0.76 (**left**) and 0.85 (**right**).

A decrease can be noted in all the evaluated metrics while maintaining significant values, even though the imbalance in classes is relevant. However, a more refined analysis shows that some models have proven to be able to properly interpret data. For example, Figure 5 shows the confusion matrices of two models, whose accuracy on $TsS_{2}$ reached 0.76 and 0.85, respectively.

### 5.2. Test2: Balanced Training Set and Unbalanced Test Set (90-30) with Holter ECGs (with Optimized Threshold and Accuracy for Best Metric)

As anticipated in the initial part of this section, in the *Test2* scenario, the *Balanced Training Set and Unbalanced Test Set* (90-30) dataset was adopted to better assess the classification performance; indeed, the use of a larger training set $TrS_{2}$ compared to the one used in the previous experiments ($TrS_{1}$) should help improve the ViT generalization capabilities. As before, the accuracy metric was adopted for monitoring the training of the models: the early-stopping technique guaranteed the conclusion of the training when the accuracy in the validation set did not improved within 10 epochs. Conversely, from the previous scenario, where a standard classification threshold (0.5) was adopted, *Test2* introduced the research of the optimized threshold, explained in Section 4.3. This process should ensure moving close to the ideal classification. At the end of each training, the generalization capability of the models with the best validation accuracy, say *bestNN_Test2*, was evaluated on $TsS_{2}$; Table 5 reports the average results of such models: it is worth noting that sensitivity and NPV are equal to 0.90 and 0.95, respectively.

### 5.3. Test3: Balanced Training Set and Unbalanced Test Set (90-30) with Holter ECGs (with Optimized Threshold and F1 for Best Metric)

The employment of accuracy as the best metric could not be the best choice if the prediction of the event ECGs is the fundamental goal, as the risk of an incorrect classification would entail serious consequences, such as patient death. In fact, accuracy works better if the cost of a false positive and a false negative is equivalent; on the contrary, the F1-score metric is more severe in penalizing incorrect prediction for *event* ECGs without neglecting the optimal classification of the *no event* class. Because a balanced validation set like $VlS_{2}$ does not guarantee that the model with the best accuracy corresponds to the model with best F1-score, additional experiments were performed, where F1-score was used for selecting the best performing models. The results are shown in Table 6. A good performance can be noted for most of the metrics, with an associated reduction in the standard deviation.

## 6. Discussion

This present work aims to present a novel application of Vision Transformer in the 12-lead ECG analysis of patients with Brugada syndrome. The reported experiments reach satisfactory results in identifying models able to analyze ECGs images and distinguish data that contain important information for the classification of patients at high risk. Table 7 shows the classification performance comparison in the different testing scenarios on the same test set $TsS_{2}$.

*Test1.1* resulted in helpful in detecting models that yielded satisfactory results, although the statistical analysis of experiments shows significant variability in the outcomes, as can be seen in Table 4.

This inconvenience is quite mitigated by the introduction of the optimal classification threshold in *Test2*, additionally supported by a consistent increase in sensitivity and NPV metrics.

By comparing Table 4 and Table 5, it can be deducted that the use of $TrS_{2}$ as a training set together with the optimized threshold mechanism boosted the performance on $TsS_{2}$, with sensitivity and NPV equal to 0.90 and 0.95, respectively.

Finally, *Test3* definitively improved the average behavior of classifications for the unbalanced test set $TsS_{2}$.

Comparing the second and third columns of Table 7 (i.e., Table 5 and Table 6), an overall increase can be noted in most metrics, with a contemporary decrease in the standard deviation, such for accuracy, PPV, specificity, F1-score, and AUC.

The high NPV and sensitivity may be used by these techniques as valuable tests for screening people at high risk of developing fatal arrhythmic events without neglecting the weighted cost that an incorrect prediction could have.

All the reported results suggest that ECG signals contain significant information for the risk stratification of patients diagnosed with Brugada syndrome. This underscores the potential of leveraging advanced techniques, such as AI-driven ECG analysis, to enhance clinical decision-making processes. By doing so, it could provide valuable support to healthcare professionals in identifying high-risk individuals, thereby contributing to the prevention of sudden cardiac death (SCD) and reducing the need for unnecessary invasive procedures, such as implantable cardioverter defibrillators (ICDs) in low-risk patients. Furthermore, AI-driven ECG analysis could help clinicians perform an early diagnosis of Brugada syndrome and could become an actionable tool for all those physicians, e.g., general practitioners, who are not familiar with the Brugada syndrome.

### Limitations of the Proposed Approach

Despite these promising results, several challenges remain. A crucial limitation is the need for a substantially larger and more diverse dataset to effectively train vision transformers and similar machine learning models, ensuring robust performance and generalizability across varied patient populations. This constraint is further compounded by the limited availability of BrS patients with documented ventricular tachycardia (VT) and SCD, which restricts the ability to comprehensively evaluate and refine these algorithms. Addressing these gaps through collaborative research and expanded data collection efforts will be essential to unlocking the full potential of AI-driven ECG analysis in the management of Brugada syndrome.

Future works will tackle these limitations by including patients from additional cohorts coming from different medical centers. Furthermore, it would be valuable to compare these results with the outcomes of electrophysiological studies, in order to assess how innovative data-driven approaches can support the standard methods currently in use.

## 7. Conclusions

An AI-enhanced ECG analysis based on vision transformer architecture is proposed for the first time as a support for the recognition of Brugada patients at a high risk of developing fatal arrhythmic events. The ECG dataset collects 94 ECGs belonging to patients with VT and SCD, while 184 ECGs are from BrS patients without arrhythmic events. In addition, a singular Holter is used for extracting 30 event ECGs added to a new test set ($TsS_{2}$).

Three different testing scenarios were considered for the classification of ECGs. For the first test (*Test1*), a balanced test dataset, $TsS_{1}$, was employed with an equal cardinality of the *event* and *no event* classes, obtaining satisfying results (accuracy of 89%, PPV of 94%, NPV of 86%, sensitivity of 84%, specificity of 94%, and an F1-score of 89%).

A fixed number of models that sum up the global distribution of the previous experimental numerical results were then tested for assessing their generalization capability on a second test set ($TsS_{2}$) composed of the unused no event ECGs (90) and 30 event ECGs extracted from the Holter recording. The consecutive testing scenarios, *Test2* and *Test3*, used the same training and validation sets derived from entire dataset of *Test1*, improving the validation process with an early stopping technique and the research of an optimized classification threshold.

At the end of the training, the models that achieved the best accuracy (in *Test2*) or F1-score (in *Test3*) on the validation set were selected and adopted for evaluating the data in the test set $TsS_{2}$. The introduction of the F1-score as a metric for selecting the best model was performed to force the training to maximize the number of true positives, even with a balanced class distribution of the validation set. It was not possible to use unbalanced training and validation sets because of the limited number of *event* BrS ECGs.

All the above techniques have proven to be efficient corrections for a consistent prediction of arrhythmic events. All the results point to the possibility that BrS ECGs contain meaningful information for the identification of people at high risk of fatal events, and AI-based ECG analysis may assist clinical decision making. However, as stated before, the number of *event* BrS ECGs should be increased to better stress and validate the proposed approach.

In conclusion, ViT has proven to be an efficient technique to deal with medical images in improving clinical diagnoses and it has been chosen as the tool to address the Brugada risk stratification task. This approach is particularly valuable, as vision transformers represent a relatively recent advancement in deep learning, showing great promise in domains traditionally dominated by convolutional neural networks. Moreover, risk stratification in Brugada patients remains an underexplored field, characterized by numerous uncertainties regarding predictive markers and clinical outcomes. Tackling this challenge with machine learning models not only underscores the novelty of this approach but also highlights its potential to provide new insights into an area with limited precedent. By leveraging the capabilities of ViT, this study paves the way for more sophisticated, data-driven methodologies to assist in managing this complex condition.

## Figures and Tables

**Table 1:** Comparison of the clinical risk parameters between the event and no-event patients and their statistical significance.

Parameter	Event (%)	No Event (%)	OR ^*a*^ (95% CI)	*p* Value
History of arrhythmic syncope	42.1	6.76	10 (4.04–25)	<0.000001
Positive SEF	70	28.4	5.88 (2–17)	0.00005
Family history of SCD < 45 years	13.1	8.78	1.57 (0.5–4.7)	0.42
SCN5A mutation	44.4	39	1.25 (0.5–3.1)	0.44
Male sex	89.4	79	2.25 (0.7–6.8)	0.14

^*a*^ OR = Odds Ratio.

**Table 2:** Report of the main metrics of *bestNN_Test1* models on $TsS_{1}$.

Metrics	Mean ^*a*^	std
Accuracy	**0.89**	0.04
PPV	**0.94**	0.07
NPV	0.86	0.05
Sensitivity	0.84	0.07
Specificity	**0.94**	0.08
F1-score	0.89	0.05

^*a*^ Most significant values are highlighted in bold.

**Table 3:** Performance comparison of the experiments of *Test1*: average behavior of 22 experiments, 10-fold cross validation, and leave-one-out. Most significant values are highlighted in bold.

Metrics	22 Experiments	10-Fold Cross Validation	Leave-One-Out
Accuracy	**0.89**	**0.87**	**0.86**
PPV	**0.94**	**0.89**	**0.88**
NPV	0.86	0.86	0.85
Sensitivity	0.84	0.85	0.85
Specificity	**0.94**	**0.89**	**0.88**
F1-score	0.89	0.86	0.86

**Table 4:** Report of the main metrics of *Test1.1* on the unbalanced test set (90-30), $TsS_{2}$.

Metrics	Mean	std
Accuracy	0.70	0.07
PPV	0.45	0.13
NPV	0.88	0.09
Sensitivity	0.68	0.32
Specificity	0.70	0.07
F1-score	0.50	0.22
AUC	0.70	0.14

**Table 5:** Report of the main metrics of *Test2* scenario on the $TsS_{2}$ test set. Most significant values are highlighted in bold.

Metrics	Mean	std
Accuracy	0.72	0.07
PPV	0.47	0.07
NPV	**0.95**	0.03
Sensitivity	**0.90**	0.07
Specificity	0.66	0.09
F1-score	0.62	0.06
AUC	0.78	0.05

**Table 6:** Report of the main metrics of *Test3* on the $TsS_{2}$ test set.

Metrics	Mean ^*a*^	std
Accuracy	0.74	0.03
PPV	0.49	0.03
NPV	**0.95**	0.03
Sensitivity	**0.90**	0.08
Specificity	0.69	0.05
F1-score	0.64	0.03
AUC	0.80	0.03

^*a*^ Most significant values are highlighted in bold.

**Table 7:** Comparison of the metrics evaluated for the prediction of $TsS_{2}$ with models generated by *Test1.1*, *Test2*, and *Test3*.

Metrics	*Test1.1*	*Test2*	*Test3*
Accuracy	0.70	0.72	0.74
PPV	0.45	0.47	0.49
NPV	0.88	0.95	0.95
Sensitivity	0.68	0.90	0.90
Specificity	0.70	0.66	0.69
F1-score	0.50	0.62	0.64
AUC	0.70	0.78	0.80

**Figure A1:**
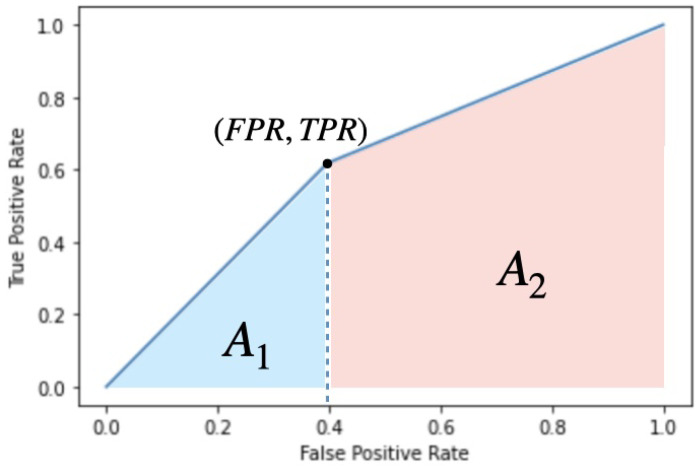
Example of ROC CURVE with a fixed threshold. The shaded area represents the AUC.

## Data Availability

The raw data supporting the conclusions of this article will be made available by the corresponding authors upon request.

## Appendix A

ROC curves are useful for assessing the performance of machine learning models. They are most commonly used for binary classification problems and depict the relationship between the model true positive rate (TPR) and the false positive rate (FPR). Usually, the generated predictive scores are continuous values within the interval [0,1]. Values greater than or equal to a certain threshold (e.g., 0.5) are classified as 1, while values below this threshold are classified as 0. The points on the ROC curve represent the FPR and TPR for different threshold values. If the threshold is fixed, the ROC curve is represented by a single point [55] with coordinates (FPR, TPR).

The predictive capabilities of models are usually compared via the AUC metric, which represents the integrated area under the ROC curve. Then, we can evaluate the total area as the sum of a triangulated area $A_{1}$ and a trapezoidal area $A_{2}$ (Figure A1).

$$\begin{array}{cc} A_{1} & =\frac{1}{2}\left(FPR\cdot TPR\right)\\ A_{2} & =\frac{1}{2}\left(1+TPR\right)\cdot \left(1-FPR\right)\\ A_{tot} & =A_{1}+A_{2}=\frac{1}{2}\left(1-FPR+TPR\right)=\frac{1}{2}\left(\frac{1-FP}{FP+TN}+\frac{TP}{TP+FN}\right) \end{array}$$

Let us denote with $P^{+}$ the number of effective positive labels (class 1) and with $N^{-}$ the negative ones (class 0). For balanced datasets, the cardinalities of the two classes are equal, then $P^{+}=N^{-}$, i.e., TP+FN=FP+TN. We can now substitute one of the two terms into $A_{tot}$ and sum the fractions:

$$\begin{array}{cc} A_{tot} & =\frac{1}{2}\left(\frac{1-FP}{FP+TN}+\frac{TP}{FP+TN}\right)\\ \; & =\frac{1}{2}\left(\frac{TN+TP}{FP+TN}\right)\\ \; & =\frac{TN+TP}{2\cdot (FP+TN)}\\ \; & =\frac{TN+TP}{TP+FN+FP+TN}=accuracy \end{array}$$

## References

[1] Brugada P., Brugada J. (1992). Right bundle branch block, persistent ST segment elevation and sudden cardiac death: A distinct clinical and electrocardiographic syndrome: A multicenter report. J. Am. Coll. Cardiol..

[2] Sieira J., Brugada P. (2017). The definition of the Brugada syndrome. Eur. Heart J..

[3] Pappone C., Santinelli V. (2019). Brugada syndrome: Progress in diagnosis and management. Arrhythmia Electrophysiol. Rev..

[4] Mizusawa Y., Morita H., Adler A., Havakuk O., Thollet A., Maury P., Wang D.W., Hong K., Gandjbakhch E., Sacher F. (2016). Prognostic significance of fever-induced Brugada syndrome. Heart Rhythm.

[5] Ohkubo K., Nakai T., Watanabe I. (2013). Alcohol-induced ventricular fibrillation in a case of Brugada syndrome. Europace.

[6] Antzelevitch C., Brugada P., Borggrefe M., Brugada J., Brugada R., Corrado D., Gussak I., LeMarec H., Nademanee K., Perez Riera A.R. (2005). Brugada syndrome: Report of the second consensus conference: Endorsed by the Heart Rhythm Society and the European Heart Rhythm Association. Circulation.

[7] Priori S.G., Wilde A.A., Horie M., Cho Y., Behr E.R., Berul C., Blom N., Brugada J., Chiang C.E., Huikuri H. (2013). HRS/EHRA/APHRS Expert Consensus Statement on the Diagnosis and Management of Patients with Inherited Primary Arrhythmia Syndromes: Document endorsed by HRS, EHRA, and APHRS in May 2013 and by ACCF, AHA, PACES, and AEPC in June 2013. Heart Rhythm.

[8] Antzelevitch C., Yan G.X., Ackerman M.J., Borggrefe M., Corrado D., Guo J., Gussak I., Hasdemir C., Horie M., Huikuri H. (2016). J-Wave syndromes expert consensus conference report: Emerging concepts and gaps in knowledge. Heart Rhythm.

[9] Richter S., Sarkozy A., Paparella G., Henkens S., Boussy T., Chierchia G.B., Brugada R., Brugada J., Brugada P. (2010). Number of electrocardiogram leads displaying the diagnostic coved-type pattern in Brugada syndrome: A diagnostic consensus criterion to be revised. Eur. Heart J..

[10] Antzelevitch C., Yan G.X., Ackerman M.J., Borggrefe M., Corrado D., Guo J., Gussak I., Hasdemir C., Horie M., Huikuri H. (2017). J-Wave syndromes expert consensus conference report: Emerging concepts and gaps in knowledge. Europace.

[11] Attia Z.I., Noseworthy P.A., Lopez-Jimenez F., Asirvatham S.J., Deshmukh A.J., Gersh B.J., Carter R.E., Yao X., Rabinstein A.A., Erickson B.J. (2019). An artificial intelligence-enabled ECG algorithm for the identification of patients with atrial fibrillation during sinus rhythm: A retrospective analysis of outcome prediction. Lancet.

[12] Paviglianiti A., Randazzo V., Villata S., Cirrincione G., Pasero E. (2022). A comparison of deep learning techniques for arterial blood pressure prediction. Cogn. Comput..

[13] Paviglianiti A., Randazzo V., Cirrincione G., Pasero E. Neural recurrent approches to noninvasive blood pressure estimation. Proceedings of the 2020 International Joint Conference on Neural Networks (IJCNN).

[14] Paviglianiti A., Randazzo V., Pasero E., Vallan A. Noninvasive arterial blood pressure estimation using ABPNet and VITAL-ECG. Proceedings of the 2020 IEEE International Instrumentation and Measurement Technology Conference (I2MTC).

[15] Wang J., Qiao X., Liu C., Wang X., Liu Y., Yao L., Zhang H. (2021). Automated ECG classification using a non-local convolutional block attention module. Comput. Methods Programs Biomed..

[16] Ge R., Shen T., Zhou Y., Liu C., Zhang L., Yang B., Yan Y., Coatrieux J.L., Chen Y. (2021). Convolutional squeeze-and-excitation network for ECG arrhythmia detection. Artif. Intell. Med..

[17] Dai H., Hwang H.G., Tseng V.S. (2021). Convolutional neural network based automatic screening tool for cardiovascular diseases using different intervals of ECG signals. Comput. Methods Programs Biomed..

[18] Liu H., Zhao Z., Chen X., Yu R., She Q. (2020). Using the VQ-VAE to improve the recognition of abnormalities in short-duration 12-lead electrocardiogram records. Comput. Methods Programs Biomed..

[19] Monedero I. (2022). A novel ECG diagnostic system for the detection of 13 different diseases. Eng. Appl. Artif. Intell..

[20] Melo L., Ciconte G., Christy A., Vicedomini G., Anastasia L., Pappone C., Grant E. (2023). Deep learning unmasks the ECG signature of Brugada syndrome. PNAS Nexus.

[21] Nakamura T., Aiba T., Shimizu W., Furukawa T., Sasano T. (2022). Prediction of the presence of ventricular fibrillation from a Brugada electrocardiogram using artificial intelligence. Circ. J..

[22] Vozzi F., Dimitri G., Piacenti M., Zucchelli G., Solarino G., Nesti M., Pieragnoli P., Gallicchio C., Persiani E., Morales M. (2022). Artificial intelligence algorithms for the recognition of Brugada type 1 pattern on standard 12-leads ECG. Europace.

[23] Liu C.M., Liu C.L., Hu K.W., Tseng V.S., Chang S.L., Lin Y.J., Lo L.W., Chung F.P., Chao T.F., Tuan T.C. (2022). A deep learning–enabled electrocardiogram model for the identification of a rare inherited arrhythmia: Brugada syndrome. Can. J. Cardiol..

[24] Sieira J., Conte G., Ciconte G., Chierchia G.B., Casado-Arroyo R., Baltogiannis G., Di Giovanni G., Saitoh Y., Juliá J., Mugnai G. (2017). A score model to predict risk of events in patients with Brugada Syndrome. Eur. Heart J..

[25] Kawada S., Morita H., Antzelevitch C., Morimoto Y., Nakagawa K., Watanabe A., Nishii N., Nakamura K., Ito H. (2018). Shanghai score system for diagnosis of Brugada syndrome: Validation of the score system and system and reclassification of the patients. Clin. Electrophysiol..

[26] Honarbakhsh S., Providencia R., Garcia-Hernandez J., Martin C.A., Hunter R.J., Lim W.Y., Kirkby C., Graham A.J., Sharifzadehgan A., Waldmann V. (2021). A primary prevention clinical risk score model for patients with Brugada syndrome (BRUGADA-RISK). Clin. Electrophysiol..

[27] Gaita F., Cerrato N., Giustetto C., Martino A., Bergamasco L., Millesimo M., Barbonaglia L., Carvalho P., Caponi D., Saglietto A. (2023). Asymptomatic patients with Brugada ECG pattern: Long-term prognosis from a large prospective study. Circulation.

[28] Gourraud J.B., Barc J., Thollet A., Le Marec H., Probst V. (2017). Brugada syndrome: Diagnosis, risk stratification and management. Arch. Cardiovasc. Dis..

[29] Priori S.G., Napolitano C., Gasparini M., Pappone C., Bella P.D., Giordano U., Bloise R., Giustetto C., De Nardis R., Grillo M. (2002). Natural history of Brugada syndrome: Insights for risk stratification and management. Circulation.

[30] Adler A. (2016). Brugada syndrome: Diagnosis, risk stratification, and management. Curr. Opin. Cardiol..

[31] Sommariva E., Pappone C., Martinelli Boneschi F., Di Resta C., Rosaria Carbone M., Salvi E., Vergara P., Sala S., Cusi D., Ferrari M. (2013). Genetics can contribute to the prognosis of Brugada syndrome: A pilot model for risk stratification. Eur. J. Hum. Genet..

[32] Monasky M.M., Micaglio E., Locati E.T., Pappone C. (2021). Evaluating the use of genetics in Brugada syndrome risk stratification. Front. Cardiovasc. Med..

[33] Asvestas D., Tse G., Baranchuk A., Bazoukis G., Liu T., Saplaouras A., Korantzopoulos P., Goga C., Efremidis M., Sideris A. (2018). High risk electrocardiographic markers in Brugada syndrome. IJC Heart Vasc..

[34] Priori S.G., Gasparini M., Napolitano C., Della Bella P., Ottonelli A.G., Sassone B., Giordano U., Pappone C., Mascioli G., Rossetti G. (2012). Risk stratification in Brugada syndrome: Results of the PRELUDE (PRogrammed ELectrical stimUlation preDictive valuE) registry. J. Am. Coll. Cardiol..

[35] Sieira J., Ciconte G., Conte G., Chierchia G.B., de Asmundis C., Baltogiannis G., Di Giovanni G., Saitoh Y., Irfan G., Casado-Arroyo R. (2015). Asymptomatic Brugada syndrome: Clinical characterization and long-term prognosis. Circ. Arrhythmia Electrophysiol..

[36] Brugada J., Brugada R., Brugada P. (2003). Determinants of sudden cardiac death in individuals with the electrocardiographic pattern of Brugada syndrome and no previous cardiac arrest. Circulation.

[37] Calò L., Giustetto C., Martino A., Sciarra L., Cerrato N., Marziali M., Rauzino J., Carlino G., De Ruvo E., Guerra F. (2016). A new electrocardiographic marker of sudden death in Brugada syndrome: The S-wave in lead I. J. Am. Coll. Cardiol..

[38] Vitali F., Brieda A., Balla C., Pavasini R., Tonet E., Serenelli M., Ferrari R., Delise P., Rapezzi C., Bertini M. (2021). Standard ECG in Brugada syndrome as a marker of prognosis: From risk stratification to pathophysiological insights. J. Am. Heart Assoc..

[39] Tse G., Lee S., Li A., Chang D., Li G., Zhou J., Liu T., Zhang Q. (2021). Automated electrocardiogram analysis identifies novel predictors of ventricular arrhythmias in Brugada syndrome. Front. Cardiovasc. Med..

[40] Probst V., Veltmann C., Eckardt L., Meregalli P., Gaita F., Tan H., Babuty D., Sacher F., Giustetto C., Schulze-Bahr E. (2010). Long-term prognosis of patients diagnosed with Brugada syndrome: Results from the FINGER Brugada Syndrome Registry. Circulation.

[41] Randazzo V., Marchetti G., Giustetto C., Gugliermina E., Kumar R., Cirrincione G., Gaita F., Pasero E. (2023). Learning-Based Approach to Predict Fatal Events in Brugada Syndrome. Applications of Artificial Intelligence and Neural Systems to Data Science.

[42] Giustetto C., Nangeroni G., Cerrato N., Rudic B., Tülümen E., Gribaudo E., Giachino D.F., Barbonaglia L., Biava L.M., Carvalho P. (2020). Ventricular conduction delay as marker of risk in Brugada Syndrome. Results from the analysis of clinical and electrocardiographic features of a large cohort of patients. Int. J. Cardiol..

[43] Saglietto A., Martinengo E., Cerrato N., Bergamasco L., Castagno D., Gaita F., De Ferrari G.M., Giustetto C. (2023). Time to positivity of diagnostic provocative pharmacologic testing in Brugada syndrome. Heart Rhythm.

[44] Zeppenfeld K., Tfelt-Hansen J., de Riva M., Winkel B.G., Behr E.R., Blom N.A., Charron P., Corrado D., Dagres N., de Chillou C. (2022). 2022 ESC Guidelines for the management of patients with ventricular arrhythmias and the prevention of sudden cardiac death: Developed by the task force for the management of patients with ventricular arrhythmias and the prevention of sudden cardiac death of the European Society of Cardiology (ESC) Endorsed by the Association for European Paediatric and Congenital Cardiology (AEPC). Eur. Heart J..

[45] Gaita F., Cerrato N., Saglietto A., Caponi D., Calò L., Giustetto C. (2023). The Brugada syndrome: Risk stratification. Eur. Heart J. Suppl..

[46] Joseph V.R. (2022). Optimal ratio for data splitting. Stat. Anal. Data Min. Asa Data Sci. J..

[47] Vaswani A., Shazeer N., Parmar N., Uszkoreit J., Jones L., Gomez A.N., Kaiser Ł., Polosukhin I. (2017). Attention is all you need. Advances in Neural Information Processing Systems.

[48] Dosovitskiy A., Beyer L., Kolesnikov A., Weissenborn D., Zhai X., Unterthiner T., Dehghani M., Minderer M., Heigold G., Gelly S. (2020). An image is worth 16x16 words: Transformers for image recognition at scale. arXiv.

[49] Chen H., Li C., Wang G., Li X., Rahaman M., Sun H., Hu W., Li Y., Liu W., Sun C. (2021). Gashis-transformer: A multi-scale visual transformer approach for gastric histopathology image classification. arXiv.

[50] Duong L.T., Le N.H., Tran T.B., Ngo V.M., Nguyen P.T. (2021). Detection of tuberculosis from chest X-ray images: Boosting the performance with vision transformer and transfer learning. Expert Syst. Appl..

[51] Wu Y., Qi S., Sun Y., Xia S., Yao Y., Qian W. (2021). A vision transformer for emphysema classification using CT images. Phys. Med. Biol..

[52] Pasero E., Gaita F., Randazzo V., Meynet P., Cannata S., Maury P., Giustetto C. (2023). Artificial Intelligence ECG Analysis in Patients with Short QT Syndrome to Predict Life-Threatening Arrhythmic Events. Sensors.

[53] Wolf T., Debut L., Sanh V., Chaumond J., Delangue C., Moi A., Cistac P., Rault T., Louf R., Funtowicz M. (2019). Huggingface’s transformers: State-of-the-art natural language processing. arXiv.

[54] Wu B., Xu C., Dai X., Wan A., Zhang P., Yan Z., Tomizuka M., Gonzalez J., Keutzer K., Vajda P. (2020). Visual transformers: Token-based image representation and processing for computer vision. arXiv.

[55] Muschelli J. (2020). ROC and AUC with a binary predictor: A potentially misleading metric. J. Classif..

